# Protest of the Alumni of Penn. College of Dental Surgery

**Published:** 1868-07

**Authors:** C. E. Latimer


### ARTICLE IX.

*Protest of the Alumni of Penn. College of Dental Surgery.*

MESSRS. EDITORS:

The following has been sent to many of the graduates of the Penn. College by a committee, and many names already received. I doubt not nearly every graduate would sign this, if we could reach them. While we bear none but the kindest feelings towards the Faculty of the Penn. College, we hope they may be induced to change their rules in the matters complained of.

Yours Truly, C. E. LATIMER.

WHEREAS the Pennsylvania College of Dental Surgery, has, within the last few years, granted diplomas to persons who have been in practice since 1852, without attending its lectures in accordance with its original rules, but has granted the degree of Doctor of Dental Surgery, in some cases we are pained to learn, without a sufficient examination,—which proceeding tends to lessen the value of the diplomas granted by the said College, both past and prospective, and

WHEREAS such a course is unjust to the regular graduates and tends to lessen the value of Dental Diplomas *generally* and thus strikes a blow at the very foundation of professional education, and

WHEREAS the said college has refused to co-operate with the association of Dental Colleges in their endeavor to elevate the standard of graduation, therefore

RESOLVED,—That we, the alumni of the Pennsylvania College of Dental Surgery, do most earnestly protest against the practices named in the above preamble, as flagrant wrong to ourselves, to our beloved Alma Mater, to the profession at large and to the people whom they serve.

